# Two Simple and Efficient Algorithms to Compute the SP-Score Objective Function of a Multiple Sequence Alignment

**DOI:** 10.1371/journal.pone.0160043

**Published:** 2016-08-09

**Authors:** Vincent Ranwez

**Affiliations:** Montpellier SupAgro, UMR AGAP, 34060, Montpellier, France; University of Michigan, UNITED STATES

## Abstract

**Background:**

Multiple sequence alignment (MSA) is a crucial step in many molecular analyses and many MSA tools have been developed. Most of them use a greedy approach to construct a first alignment that is then refined by optimizing the sum of pair score (SP-score). The SP-score estimation is thus a bottleneck for most MSA tools since it is repeatedly required and is time consuming.

**Results:**

Given an alignment of n sequences and L sites, I introduce here optimized solutions reaching *O*(*nL*) time complexity for affine gap cost, instead of *O*(*n*^2^*L*), which are easy to implement.

## Introduction

A wide range of molecular analyses rely on multiple sequence alignments (MSA), e.g., prediction of tridimensional structures [1], phylogenetic inference [2] or detection of positive selection [3]. In all these studies, the initial MSA can strongly impact conclusions and biological interpretations [4,5]. As a consequence, MSA is a richly developed area of bioinformatics and computational biology.

Most MSA software use a greedy approach to construct a first alignment that is then refined by optimizing the sum of pair score (SP-score) [6,7] using a 2-cut strategy. This strategy consists in partitioning the current solution into two sub-alignments that are subsequently re-aligned; the resulting MSA replaces the previous one if its SP score is improved [7,8,9,10]. The SP-score estimation is thus a bottleneck for most MSA tools since it is repeatedly required and is time consuming as existing solutions have a time complexity of *O*(*n*^2^*L*) for an alignment of *n* sequences and *L* sites. The practical importance of having an efficient solution to compute SP-score lies within the following ‘Muscle software paper’ [8] quotation: “Notice that computation of the SP score dominates the time complexity of refinement and of MUSCLE overall[…]. It is natural to seek an *O*(*nL*) expression for [SP-score estimation…], but to the best of our knowledge no solution is known.”

The main result of this paper is an optimized algorithmic solution to estimate SP-score for affine gap costs in *O*(*nL*). I also introduce a more versatile solution able to handle more general gap cost penalty functions in *O*(*nL* + *n*^2^*G*_*max*_), with *G*_*max*_ ≤ *L* being the maximum number of gap intervals within one aligned sequence.

## Materials and Methods

### Definitions and notations

A multiple sequence alignment (MSA) for a set of sequences {*s*_*1*_…*s*_*n*_}, defined with alphabet Σ, is a set of *n* sequences {*S*_*1*_…*S*_*n*_} which are defined on an enriched alphabet Σ ∪{‘-’} so that all *S*_i_ have the same length *L* and, ∀*i*, removing ‘-’ from *S*_*i*_ leads to *s*_*i*_. The aim of MSA tools is to position gaps (stretches of ‘-’) so that characters at the same position (constituting a site) are (most likely) homologous. This is usually achieved through a heuristic optimization of the sum of pair score (SP-score). The SP-score of an MSA is obtained by considering all pairwise alignments it induced. Given two sequences *S*_*i*_ and *S*_*j*_ of MSA A the corresponding pairwise restriction (A |{*S*_*i*_,*S*_*j*_}) is the alignment made up of two sequences $S_{i}^{\prime }$ and $S_{j}^{\prime }$ obtained by removing the ‘-’ of *S*_*i*_ (resp. *S*_*j*_) whenever *S*_*j*_ (resp. *S*_*i*_) also has a gap at this position/site. The score of this pairwise alignment is the sum of the substitution/match scores induced by sites without gaps, plus the sum of scores associated to the gap intervals (maximal sub-sequences of consecutive gap symbols) observed in $S_{i}^{\prime }$ and $S_{j}^{\prime }$.

The SP-score of an alignment is classically obtained in *O*(*n*^2^*L*) by summing up the score of its (n2) induced pairwise alignments (Algorithm 1 and 2), and can be decomposed into two terms: SPs, the contribution of substitutions/matches and, SPg the contribution of induced gap intervals (denoted IG’).

In molecular biology |Σ| is a constant so typically small (4 for nucleotides and 20 for amino acids) that *L*|Σ|^2^ and *n*|Σ|^2^, compared to *nL*, can safely be ignored in asymptotic complexity analysis. Under this assumption, all solutions described here have an *O*(*nL*) space complexity.

**Algorithm. 1**. Basic *O*(*n*^2^*L*) computation of the SP-score of an alignment A of *n* sites and *L* sequences given subst(.,.) and g_cost(.) functions. The subst(.,.) function provides, in *O*(1), the elementary score for two non gap characters on the same site, e.g. using BLOSUM matrix [11]. The g_cost(.) function provides, also in *O*(1), the cost of a gap interval based on its position and size. The compute_gap_intervals(.) subroutine (Algorithm 2) returns in *O*(|*S*|) the list of the gap intervals of its input sequence *S*.

Algorithm 1: compute_SP_score

Input: -The *n* aligned sequences *S*_*i*_ of alignment A

        -a function subst(x,y) returning in *O(1)* the score for two non gap characters x and y on the same site of A

        -a function g_cost(IG’) returning in *O(1)* the cost of a gap interval IG’

Output: the SP score of this alignment

SP_s_ = 0; SP_g_ = 0

for *S*_*i*_ in *S*_1_ … *S*_*n*_

    for *S*_*j*_ in *S*_*i*+1_ … *S*_*n*_

        $S_{i}^{\prime }=S_{j}^{\prime }=""$

        for *k* in 1…L

            if(not (*S*_*i*_[*k*] == ‘–’ *and S*_*j*_[*k*] == ‘–’))

                $S_{i}^{\prime }=S_{i}^{\prime }+S_{i}[k]$

                $S_{j}^{\prime }=S_{j}^{\prime }+S_{j}[k]$

            if(*S*_*i*_[*k*] ≠ ′–′ and *S*_*j*_[*k*] ≠ ′–′)

                SP_s_ = SP_s_ + subst($S_{i}^{\prime }[k],S_{j}^{\prime }[k]$)

        for IG’ in compute_gap_intervals($S_{i}^{\prime }$) ∪ compute_gap_intervals($S_{j}^{\prime }$)

            SP_g_ = SP_g_ + g_cost (IG’) // e.g., g_cost(IG’) = {return gap_O_ + IG’[length]*gap_ext_}

Return SP_s_ + SP_g_

**Algorithm. 2**. An *O*(*L*) algorithm to compute the list of gap intervals, ordered by their gap start position, of a sequence *S* of length *L*. Note that, thought IG[length] is not explicitly set, it is assumed it can be access since *IG*[length] is simply *IG*[end]-*IG*[start]+1.

Algorithm 2: compute_gap_intervals

Input: An aligned sequences *S*_*i*_ of length *L*

Output: The list of gap intervals of *S*_*i*_, ordered by gap start position

*LG* = {}; // the list of gap intervals of *S*_*i*_ found so far

*IG* = NULL; // the current gap interval

for *k* in 1…*L*

    if(*S*_*i*_[*k*] == ′–′ and *IG* = = NULL) // start a new gap interval

        *IG* = new Interval(start = k)

    if(*S*_*i*_[*k*] ≠ ′–′ and *IG* ≠ NULL) // the current gap interval finish at previous position

        *IG*[end] = k-1; append a copy of *IG* to the list *LG*

        *IG* = NULL

if (*IG* ≠ NULL) // handle terminal gap if any

        *IG*[end] = L; append a copy of *IG* to the list *LG*

Return *LG*

### Efficient algorithm to compute SP-score using general gap cost penalties

The SPs part of the SP-score can be computed in O(*nL*) by using a table of size *L*|Σ| containing for each site the number of each (non gap) symbol (e.g. [8]). This strategy does not work for SPg except for the basic, but unrealistic, constant gap cost where g_cost(IG’) = IG’[length].gapcost. I introduce here a more efficient solution to the SP-score computation problem accounting for most gap function penalties (including affine, log, log-affine penalties). The main idea is to pre-compute the list of gap intervals of each sequence *S*_*i*_, ordered by gap start position, this can easily be done in *O*(*L*) using Algorithm 2. The compute_gap_intervals($S_{i}^{\prime }$) and compute_gap_intervals($S_{j}^{\prime }$) of Algorithm 1, observed in A|{*S*_*i*_,*S*_*j*_}, can then be efficiently deduced by processing the gaps of compute_gap_intervals(*S*_*i*_) ∪ compute_gap_intervals(*S*_*j*_) according to order of opening (as done to merge two sorted lists in linear time, e.g. during a merge step of the ‘merge sort’ algorithm) while maintaining the number of gaps facing gaps encountered so far (i.e. the shift between *S* and *S’* site coordinate systems for current position). The resulting SP-score algorithm (Algorithm 3) has a complexity of *O*(*nL* + *n*^2^*G*_*max*_), with *G*_*max*_ ≤ ⌈*L*/2⌉ the maximum number of gap intervals within one aligned sequence, instead of *O*(*n*^2^*L*). Note that the difference with the naïve algorithm is especially important when sequences contain few long gap stretches but that in the worst case, where most sequences have a number of gap intervals close to *L*, this algorithm has the same *O*(*n*^2^*L*) complexity as the naïve solution.

### Optimal algorithm to compute SP-score using affine gap cost penalties

Affine gap penalties (where g_cost(IG’) = gap_O_ + IG’[length].gap_ext_) are frequently used. For such gap penalties, the total of gap extension penalties (SP_ge_) can also be efficiently computed in *O*(*nL*), by counting the number of gaps per site. However, gap opening cannot be counted exactly based on local site information (e.g [8]) only approximated. Though pessimistic gap count approximation [9] is often used during the dynamic programming steps producing new candidate alignments, the exact SP score is generally preferred to decide which alignments are better than the current one. Algorithm 4 provides a simple and exact solution to compute the SP-score under affine gap penalties in *O*(*nL*), which is also the time complexity for just reading an alignment of *n* sequences of *L* sites.

The key idea is to note that a gap *IG*_*i*_ will add a number of gap opening penalties equal to *n* minus the number of interval *IG*_*j*_ so that *IG*_*i*_ ⊆ *IG*_*j*_. In order to find out how many gaps encompass *IG*_*i*_, sites are processed from left to right while maintaining an array indicating, for each left site, the number of gap stretches already opened at this position and not yet closed. For all gaps *IG*_*i*_ closing at the current position *i*, the value stored at index *IG*_*i*_[*deb*] of this array provides, in *O*(1), the number of gap stretches encompassing *IG*_*i*_; before considering site *i*+1, this array is maintained updated by decreasing by 1 all values stored at indices between *IG*_*i*_[*deb*] and *i* for all *IG*_*i*_ ending at *i*—hence updates for all sites overall require *O*(*nL*). External and internal gaps are often penalized with different affine functions. The proposed *O*(*nL*) solution can handle this refinement by: firstly, using different characters (e.g. ‘-’ and ‘_’) to represent the two different gap types while computing SP_ge_; and secondly, testing each gap interval type in Algorithm 3 (using gap interval start/end positions) to select the adequate gap opening cost.

**Algorithm. 3**. Given the gap interval lists *LG*_*i*_, *LG*_*j*_ of sequences $S_{i}\in A$ and $S_{j}\in A$; this algorithm returns in *O*(|*LG*_*i*_| + |*LG*_*j*_|) the restricted gap interval lists ${LG}_{i}^{\prime }$, ${LG}_{j}^{\prime }$ that would be observed in A|{*S*_*i*_,*S*_*j*_} without actually building this restricted alignment.

Algorithm 3: compute_pairwise_restricted_gap_intervals

Input:—*LG*_*i*_, *LG*_*j*_ the ordered lists of gap intervals for $S_{i}\in A$ and $S_{j}\in A$

Output:—${LG}_{i}^{\prime }$, ${LG}_{j}^{\prime }$ the lists of gap intervals in A|{*S*_*i*_,*S*_*j*_}

*IG*_*i*_ = first(*LG*_*i*_); *IG*_*j*_ = first(*LG*_*j*_)

shift = 0;

${LG}_{i}^{\prime }={LG}_{j}^{\prime }$ = {}

${IG}_{i}^{\prime }=$ new Interval(start = -1)

${IG}_{j}^{\prime }=$ new Interval(start = -1) // using -1 allows to check if interval start has already been set or not

while(*IG*_*i*_≠NULL and *IG*_*j*_≠NULL)

    if(*IG*_*i*_[start] == *IG*_*j*_[start])

            if(*IG*_*i*_ == *IG*_*j*_) // both intervals disappear when A is restricted to A|{*S*_*i*_,*S*_*j*_}

                *IG*_*i*_ = next(*LG*_*i*_); ${IG}_{i}^{\prime }=$ new Interval(start = -1)

                *IG*_*j*_ = next(*LG*_*j*_); ${IG}_{j}^{\prime }=$ new Interval(start = -1)

            elif(*IG*_*j*_ ⊂ *IG*_*i*_)//*IG*_*j*_ disappear during restriction

                *IG*_*j*_ = next(*LG*_*j*_); ${IG}_{j}^{\prime }=$ new Interval(start = -1)

                if(${IG}_{i}^{\prime }$[start] = = -1) //${IG}_{i}^{\prime }$[start] is now known

                    ${IG}_{i}^{\prime }$[start] = *IG*_*i*_[start]-shift

            else // (*IG*_*i*_⊂ *IG*_*j*_) // *IG*_*i*_ disappear during restriction

                ……. // similar to previous case swapping *i* and *j*

            shift = shift+|*IG*_*i*_ ∩ *IG*_*j*_|

    elif(*IG*_*i*_[start]<*IG*_*j*_[start])

        if(*IG*_*j*_ ⊂ *IG*_*i*_) // *IG*_*j*_ disappear during restriction, shift increase

            if(${IG}_{i}^{\prime }$[start] = = -1) // set ${IG}_{i}^{\prime }$[start], if not already done, before increasing shift

                ${IG}_{i}^{\prime }$[start] = *IG*_*i*_[start]-shift

        shift = shift+|*IG*_*i*_ ∩ *IG*_*j*_|

        *IG*_*j*_ = next(*LG*_*j*_); ${IG}_{j}^{\prime }=$ new Interval(start = -1)

    else // *IG*_*j*_ start after *IG*_*i*_ and is not included in *IG*_*i*_

        if(${IG}_{i}^{\prime }$[start] = = -1)

            ${IG}_{i}^{\prime }$[start] = *IG*_*i*_[start]-shift

        if(*IG*_*i*_ ∩ *IG*_*j*_≠⊘) //${IG}_{j}^{\prime }$[start] is now known and shift increase

            ${IG}_{j}^{\prime }$[start] = *IG*_*j*_[start]-shift

            shift = shift + |*IG*_*i*_ ∩ *IG*_*j*_|

        ${IG}_{i}^{\prime }$[end] = *IG*_*i*_[end]-shift

        append ${IG}_{i}^{\prime }$ to ${LG}_{i}^{\prime }$

        *IG*_*i*_ = next(*LG*_*i*_); ${IG}_{i}^{\prime }=$ new Interval(start = -1)

    else // (*IG*_*j*_[start]<*IG*_*i*_[start])

        ……. // similar to previous case swapping *i* and *j*

if(*IG*_*i*_≠NULL) // handle last gaps in *LG*_*i*_

    if(${IG}_{i}^{\prime }$[start] = = -1){${IG}_{i}^{\prime }$[start] = *IG*_*i*_[start]-shift}

    ${IG}_{i}^{\prime }$[end] = *IG*_*i*_[end]-shift

    append ${IG}_{i}^{\prime }$ to ${LG}_{i}^{\prime }$

    while((*IG*_*i*_ = next(*LG*_*i*_) ≠ NULL)

        append new Interval(start = *IG*_*i*_[*start*]-shift; end = *IG*_*i*_[*end*]-shift)to ${LG}_{i}^{\prime }$

if(*IG*_*j*_≠NULL)

        …… //similar to previous block replacing *i* with *j*

return ${LG}_{i}^{\prime }$, ${LG}_{j}^{\prime }$

## Conclusion

This paper introduces an optimized algorithmic solution to estimate SP-score for affine gap costs in *O*(*nL*) and a more versatile solution able to handle more gap cost penalty functions in *O*(*nL* + *n*^2^*G*_*max*_), with *G*_*max*_ ≤ ⌈*L*/2⌉ being the maximum number of gap intervals per sequence. These optimizations will obviously be part of the next release of MACSE [10], the MSA software we developed to align nucleic sequences with respect to their amino acid translation while allowing them to contain frameshifts and/or stop codons (http://bioweb.supagro.inra.fr/macse/). Moreover, once stated those two algorithms are quite straightforward and can easily be included in the numerous existing MSA software relying on SP-score.

**Algorithm. 4. **Efficient *O*(*nL*) computation of the contribution of gap opening cost (*SP*_*go*_) for an alignment A of n sites and *L* sequences.

Algorithm 4: compute_SP_go__using_gap_intervals

Input:—the *n* aligned sequences *S*_1_…*S*_*n*_ of A

        - the costs of a gap opening within a sequence (gap_O_) or at its extremities (gap_O_ext_)

Output: SP_go_: the part of the SP-score of A due to gap opening costs

nbOpenGap = new Array of *L* integers initialized to 0;

gapClosing = new Array of *L* empty lists of Intervals;

for *S*_*i*_ in *S*_1_…*S*_*n*_

    //construct *LG*_*i*_ in *O*(*L*) and update nbOpenGap and gapClosing arrays

    *LG*_*i*_ = compute_gap_intervals(*S*_*i*_)

    foreach *IG*_*i*_ of *LG*_*i*_

        for *k* in *IG*_*i*_[start]…*IG*_*i*_[end]

            nbOpenGap[k]++

    append *IG*_*i*_ to gapClosing[*IG*_*i*_[end]]

SP_go_ = 0; // part of the SP score related to gap opening costs

for *i* in 1 … *L*

    foreach *IG*_*i*_ in gapClosing[*i*]

        if(*i* == *L* OR *IG*_*i*_[start] = = 1)

            SP_go_ = SP_go_+(n-nbOpenGap[*IG*_*i*_[start]])* gap_O_ext_

        else

            SP_go_ = SP_go_+(n-nbOpenGap[*IG*_*i*_[start]])* gap_O_

    foreach *IG*_*i*_ in gapClosing[*i*]

        for *k* in *IG*_*i*_[start] … *IG*_*i*_[end]

            nbOpenGap[*k*] = nbOpenGap[*k*]-1;

return SP_go_
